# Navigating the future of gastric cancer treatment: a review on the impact of antibody-drug conjugates

**DOI:** 10.1038/s41420-025-02429-5

**Published:** 2025-04-05

**Authors:** Qingling Yin, Yanlong Zhang, Xueqing Xie, Meijun Hou, Xunsheng Chen, Jie Ding

**Affiliations:** 1 GuiZhou University Medical College, Guiyang, 550025 Guizhou China; 2 NHC Key Laboratory of Pulmonary Immunological Diseases, Guizhou Provincial People’s Hospital, Guiyang, 550002 Guizhou China; 3 Graduate School, Zunyi Medical University, Zunyi, Guizhou 563006 China; 4 Department of Gastrointestinal Surgery, Guizhou Provincial People’s Hospital, Guiyang, 550002 Guiyang, China

**Keywords:** Cancer therapy, Drug delivery

## Abstract

Gastric cancer, marked by its high incidence and poor prognosis, demands the urgent development of novel and effective treatment strategies, especially for patients ineligible for surgery or those who have had limited success with chemotherapy, radiotherapy and targeted therapies. Recently, antibody-drug conjugates (ADCs) have become a key area of investigation due to their high specificity and potent antitumor effects. These therapies combine monoclonal antibodies, designed to bind to tumor-specific antigens, with cytotoxic agents that selectively target and destroy malignant cells. ADCs have generated significant interest in clinical trials as a promising approach to improve both treatment efficacy and patient outcomes in gastric cancer. However, their clinical application is not without challenges and limitations that must be addressed. This review discusses the recent progress in the use of ADCs for gastric cancer treatment.

## Facts

- Antibody-drug conjugates have gained considerable attention in research for their high selectivity and potent antitumor activity.
- Evolution of gastric cancer involves multiple mechanisms.
- Continued research and development of antibody-drug conjugates must be supported by comprehensive clinical data and accurate disease classification to ensure their effective integration into clinical practice.

## Questions

- What molecular mechanisms drive the pathogenesis of gastric cancer?
- How do antibody-drug conjugates modulate the tumor microenvironment to exert their effects?
- What are the clinical outcomes associated with antibody-drug conjugates, and what implications do they hold for patient care?
- How can the design and optimization of antibody-drug conjugates be advanced to improve their efficacy and safety?

## Introduction

**Fig. 1:**
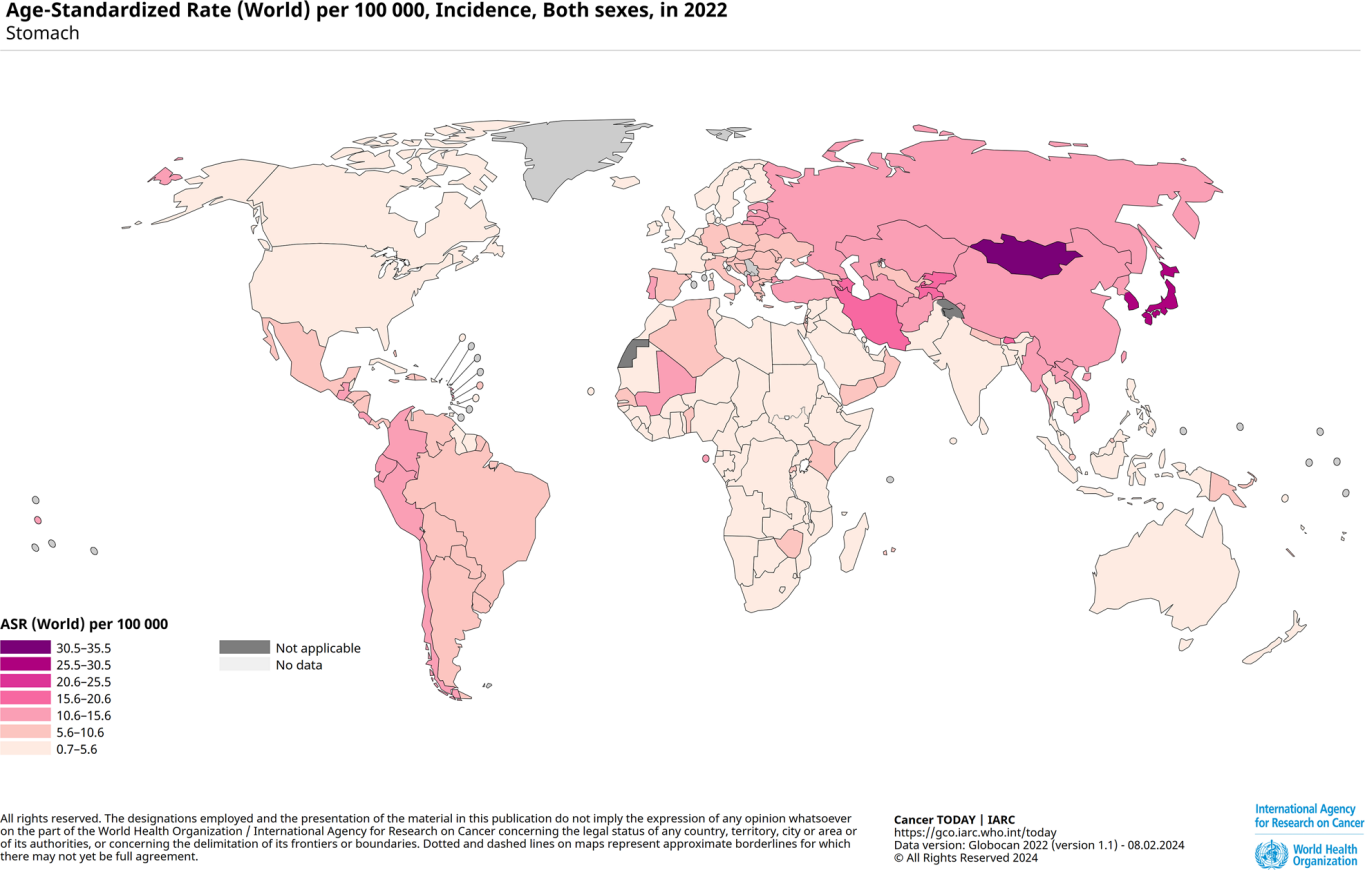
Global maps present the seven-tier Age-Standardized Rate per 100000. The sizes of the respective populations are included in the legend. Source: United Nations Procurement Division/United Nations Development Program. ASR indicates Age-Standardized Rate. Source: GLOBOCAN 2022.

**Fig. 2:**
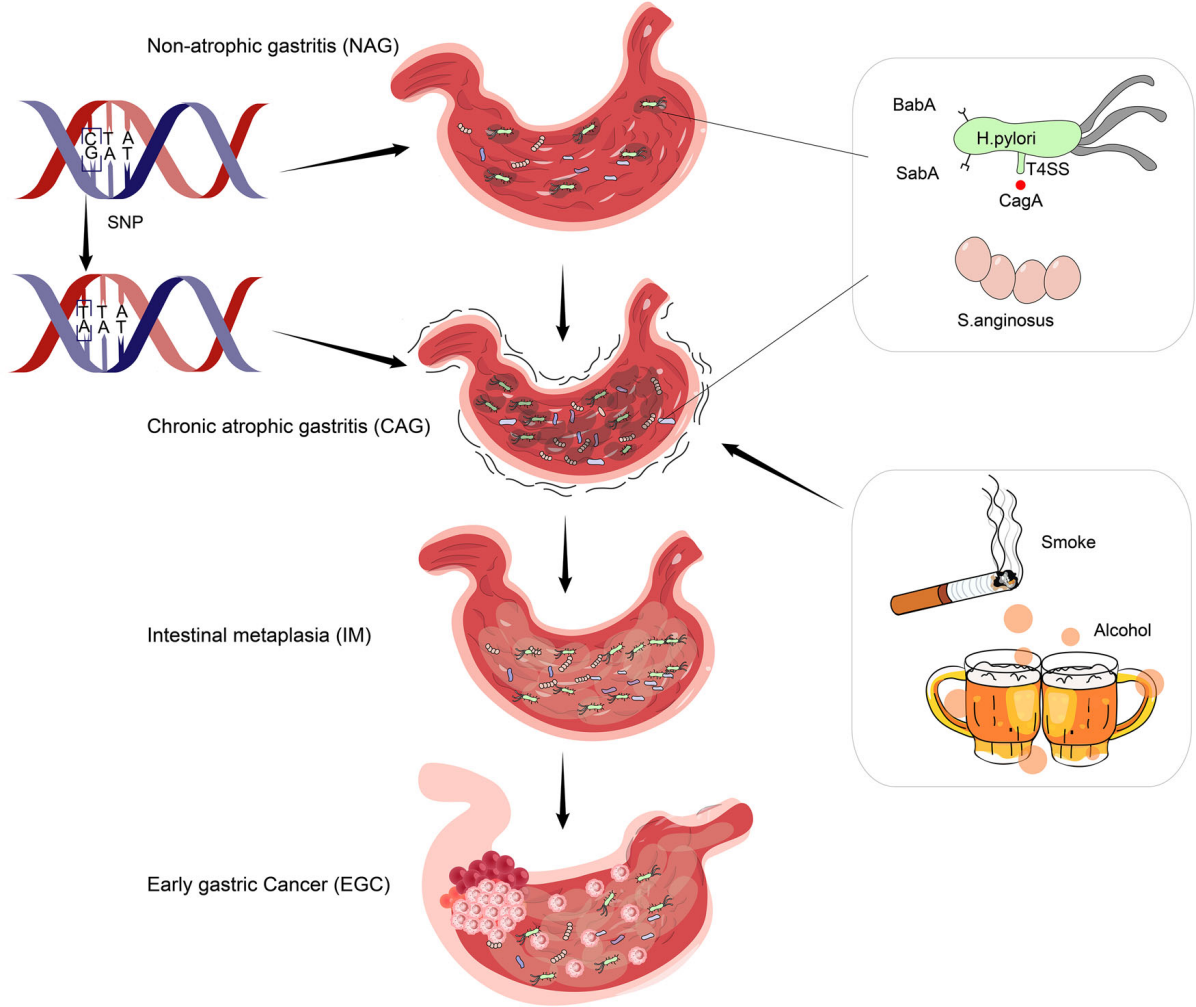
Evolution of GC and its causes. The progression from common gastric lesions to GC occurs through four stages: Stage 1: Chronic non-atrophic gastritis. Stage 2: Chronic atrophic gastritis, a precancerous lesion that warrants attention. In cases of Helicobacter pylori infection, eradication should be initiated without delay. Stage 3: Intestinal metaplasia, a further advancement in precancerous lesions. If left untreated, some of these lesions may progress to early GC. Stage 4: GC, the final malignant stage, typically presents with no distinct symptoms or signs in its early phases. The etiology of GC is multifactorial, including lifestyle, dietary habits, infectious agents, genetic predispositions, and other contributing factors.

Gastric cancer (GC) ranks as the fifth most prevalent malignancy globally and is the third leading cause of cancer-related mortality [1], reflecting the substantial health burden it imposes worldwide (Fig. 1). Over 95% of GC cases are adenocarcinomas originating from the gastric mucosal epithelium. These adenocarcinomas are categorized based on their anatomical location (cardia/proximal or non-cardia/distal) and histological subtype (diffuse or intestinal) [2]. The diffuse type of GC is characterized by poorly differentiated, scattered tumor cells with irregular or absent glandular structures, along with diffuse infiltration into the gastric lining and proliferative mesenchymal connective tissue. This subtype is more commonly observed in low-risk regions and is predominantly linked to genetic mutations, indicating a significant genetic predisposition [3]. In contrast, the intestinal type of GC is marked by tumor cells that exhibit variable differentiation and form tubular or glandular patterns, interspersed with scattered cup-shaped cells. It is the predominant form in high-risk areas and accounts for much of the geographic variability in GC incidence. The intestinal type is strongly associated with environmental risk factors, including Helicobacter pylori (H. pylori) infection, smoking, and high salt intake, highlighting the influence of lifestyle and dietary habits on its development (Fig. 2) [3, 4]. Certain GC cases also present TP53 gene mutations, often accompanied by distinct morphological changes [5]. while a significant correlation has been observed between H. pylori infection and kirsten rat sarcoma viral oncogene (KRAS) mutations in GC patients [6]. These mutations may serve as important diagnostic markers, guiding the treatment of various malignancies.

Surgical interventions are effective in managing early-stage GC, while advanced-stage cases necessitate a multifaceted approach, which may include surgery, perioperative chemotherapy, radiotherapy, targeted therapy, and immunotherapy [1]. The role of biologics in first-line treatment for advanced GC is currently under investigation, particularly in relation to trastuzumab [1]. Treatment strategies are tailored to the patient’s specific pathology and clinical stage. Although treatment options for GC have advanced significantly in recent years, the majority of patients are diagnosed at later stages, restricting curative treatment possibilities. This is largely attributed to the high incidence and asymptomatic nature of the disease [7, 8]. Consequently, a deeper understanding of the molecular mechanisms underlying GC pathogenesis and metastasis is essential to identify novel therapeutic targets that could enhance patient outcomes.

For patients with intermediate and advanced GC, the integration of targeted therapies based on molecular targets marks a significant advancement in the precision surgical treatment of GC [9]. Targeted therapy has become a key innovation in cancer management, operating on the principle of developing drugs that intervene at the molecular level within cells. These therapeutics are specifically designed to interact with defined molecular sites within tumor cells that drive oncogenesis, such as particular proteins or gene fragments. Upon administration, these drugs selectively bind to their targets, leading to the targeted destruction of tumor cells while sparing surrounding healthy tissues [10, 11]. Despite their effectiveness, targeted therapies have limitations, as patients may experience adverse effects and develop resistance. Cancer treatment resistance is typically classified into primary and secondary types. Primary resistance refers to the absence of any initial therapeutic response, signifying that the treatment is ineffective from the start. Secondary or acquired resistance occurs when an initially responsive treatment loses its efficacy over time, allowing tumor progression to resume [12]. Thus, the identification of novel therapeutic targets and approaches remains essential, not only to address treatment-resistant tumors but also to counteract drug resistance, ultimately enhancing patient outcomes. Such efforts are critical to the continued advancement of cancer treatment [12, 13].

Recent advancements in monoclonal antibody technology, coupled with innovations in payloads, linkers, and tumor-specific targets, have led to the emergence of antibody-drug conjugates (ADCs) as a novel class of therapeutic agents [14]. ADCs have become a focal point in cancer drug development, offering a new strategy for treatment [15]. These conjugates integrate antibodies that selectively bind to antigens on cancer cells with cytotoxic drugs capable of inducing cell death. This targeted approach enables the precise delivery of the cytotoxic agent to cancer cells, minimizing collateral damage to healthy tissue [16]. Often referred to as “bio-missiles” or “magic bullets,” ADCs are celebrated for their precision in drug delivery. The concept of “magic bullets,” first introduced by German immunologist Paul Ehrlich, envisioned monoclonal antibodies (mAbs) capable of selectively targeting pathogens without harming the host organism. The progression of ADC development has been greatly enhanced by advances in chemical conjugation techniques and the production of humanized mAbs, facilitating their approval for clinical trials [17]. As of the end of 2022, 15 ADCs have received regulatory approval for clinical use worldwide (Table 1), marking a significant milestone in cancer therapeutics.

**Table 1:** Globally approved and marketed antibody-drug conjugates.

Trade name	Name of drug	Research and development organization	Date of first listing	Therapeutic target	Type of cancer
Mylotarg	Gemtuzumab Ozogamicin	Pfizer, American pharmaceutical company	2000/05/17 (first authorized) 2010/06 (withdrawn from the market) 2017/09/01 (reauthorized)	CD33	Leukaemia
Adcetris	Brentuximab Vedotin	SEAGEN/Takeda	2011/08/19	CD30	Hodgkin’s lymphoma, Mesenchymal large cell lymphoma
Kadcyla	Trastuzumab Emtansine	F. Hoffmann-La Roche Ltd	2013/02/22	HER2	HER2-positive breast cancer
Besponsa	Inotuzumab Ozogamicin	Pfizer, American pharmaceutical company	2017/06/28	CD22	B-cell acute lymphoblastic leukemia
Lumoxiti	Moxetumomab Pasudotox	AstraZeneca	2018/09/13	CD22	Capillary leukemia
Polivy	Polatuzumab Vedotin	F. Hoffmann-La Roche Ltd	2019/06/10	CD79b	Diffuse large B-cell lymphoma
Padcev	Enfortumab Vedotin	SEAGEN/Anstellar	2019/12/18	Nectin-4	Urothelial carcinoma
Enhertu	Trastuzumab Deruxtecan	Daiichi Sankyo/AstraZeneca	2019/12/20	HER2	HER2-positive breast cancer
Trodelvy	Sacituzumab Govitecan	Gilead, an American pharmaceutical company	2020/04/22	Trop-2	Triple-negative breast cancer
Blenrep	Belantamab Mafodotin	GSK	2020/08/05 (first approved) 2022/11/22 (withdrawn from the market)	BCMA	Multiple myeloma
Akalux	Cetuximab Saratolacan	Rakuten Medical	2020/09/25	EGFR	Head and neck cancer
Zynlonta	Loncastuximab Tesirine	ADC Therapeutics	2021/04/23	CD19	B-cell lymphoma
Disitamab Vedotin	Vidicilimumab	Rongchang Bio	2021/06/08	HER2	Gastric cancer
Tivdak	Tisotumab Vedotin	Genmab/Seagen	2021/09/20	TF	Cervix
Elahere	Mirvetuximab Soravtansine	ImmunoGen/Donghua Pharmaceuticals	2022/11/14	FRα	Epithelial ovarian, Fallopian tube, Primary peritoneal cancer

*HER2* human epidermal growth factor receptor 2, *TROP2* trophoblast cell surface antigen 2, *BCMA* B cell maturation antigen, *EGFR* epidermal growth factor receptor, *TF* tissue factor, *FRα* folate receptor alpha.

Over the past decade, substantial progress has been made in the development of ADCs [18], driven by the identification of more potent cytotoxic agents, the refinement of bio-conjugation techniques, improved antigen targeting, and advancements in antibody engineering. However, ADCs continue to encounter significant challenges, including limited tumor penetration, toxicity, and the emergence of drug resistance. To overcome these obstacles, research has concentrated on novel antibody formats, innovative delivery systems, non-internalizing targets, novel cytotoxic agents, and precise site-specific conjugation strategies. Currently, over 100 ADC candidates are undergoing clinical evaluation [18]. Although many of these innovations remain unvalidated in clinical practice, preliminary research has produced promising results. This review aims to provide an overview of the latest developments in ADC technology, address current challenges and unresolved issues, and explore potential future directions in this rapidly advancing field.

## Antibody-drug conjugate

### Components

**Fig. 3:**
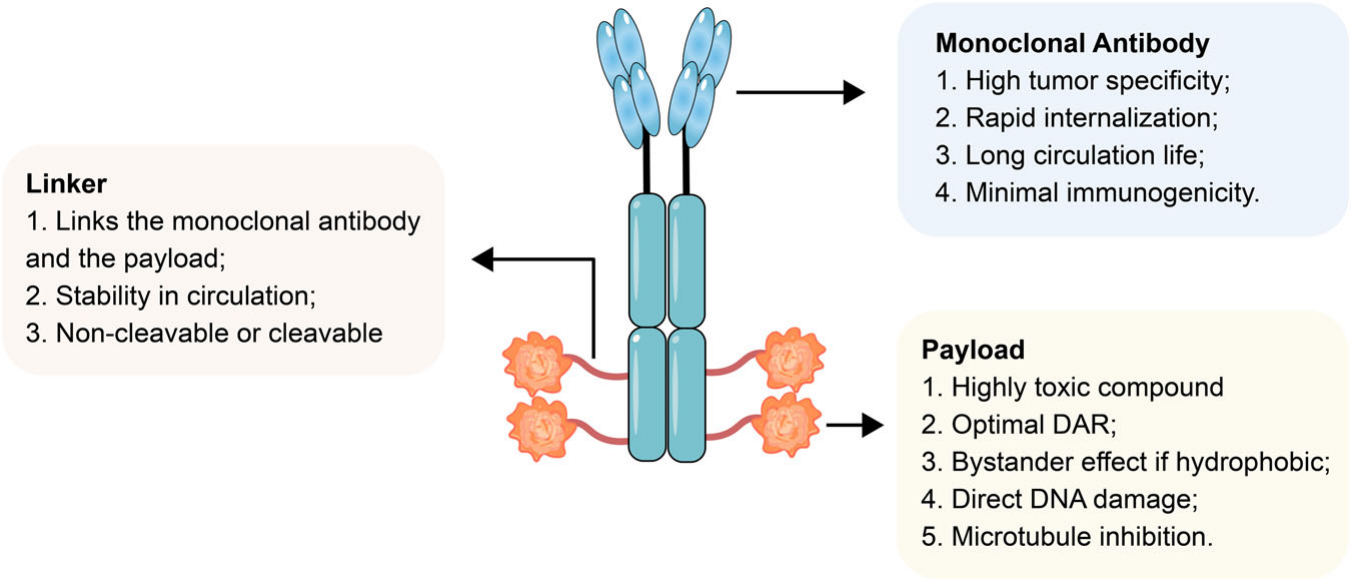
Antibody-drug conjugate (ADC) structure and key properties. ADC is a complex structure composed of three key components. These include the antibody, the cytotoxic payload, and the linker that connects them, with each part playing an essential role.

An ADC consists of three essential components: an antibody, a cytotoxic payload and a linker (Fig. 3). The antibody is engineered to specifically bind to target molecules expressed on tumor cells. Upon binding, the cytotoxic payload is delivered, resulting in the selective destruction of the tumor cells, thereby enabling targeted cancer therapy [19].

#### Recombinant mAbs

The mAbs are immunoglobulins with high specificity (mono-specificity) for a particular antigen or epitope [20]. Initially, antibodies used in ADC development were derived from mice. However, their high failure rates were attributed to severe side effects caused by immunogenicity, which refers to an antigen’s ability to provoke an immune response. This response is triggered by the antigen, leading to the activation, proliferation, differentiation, and production of antibodies and sensitized lymphocytes by specific immune cells [21]. With the introduction of recombinant technology, murine antibodies were gradually replaced by chimeric and humanized antibodies, resulting in reduced immunogenicity [22, 23]. The majority of ADCs now employ fully humanized antibodies, further minimizing immunogenicity. Among the 15 approved ADCs (Table 1), only Brentuximab vedotin utilizes chimeric antibodies [24]. Most ADCs incorporate immunoglobulin G (IgG) as the antibody backbone, which consists of four subtypes: IgG1, IgG2, IgG3, and IgG4 [18], all of which are key components of serum immunoglobulins. Humanized mAbs effectively reduce ADC immunogenicity and side effects. Subtle differences among these IgG subclasses can impact mAb solubility, half-life, and affinity for various Fcγ receptors (FcγR) expressed in immune effector cells [25, 26]. IgG1, which shares a long serum half-life with IgG2 and IgG4, is the most abundant IgG subtype in serum. However, compared to other IgG subclasses, IgG1 binds more efficiently to the FcγR, making it the preferred subtype for ADCs [27].

Recombinant mAbs play a vital role in mediating antitumor effects by specifically targeting antigens on tumor cells. This enables the precise delivery of cytotoxic drug payloads directly to the tumor site [23]. However, antibody production, storage, and transportation may induce modifications that compromise their structural integrity and biological activity, thereby affecting the efficacy of ADCs [18]. An ideal monoclonal antibody should exhibit a high binding affinity for the target antigen, promote efficient internalization, have low immunogenicity, and maintain a prolonged plasma half-life [28]. The internalization efficiency of ADCs is largely influenced by the affinity between the antibody and the tumor cell surface antigen. While increased affinity typically accelerates internalization, excessively high-affinity antibodies may limit penetration into solid tumors [28, 29]. Thus, optimizing the affinity between the antigen and antibody is critical during drug development to ensure effective cellular uptake and enhanced anticancer activity.

#### Cytotoxic Payload

Cytotoxic payloads, which are small-molecule drugs, are engineered to selectively eliminate tumor cells [30]. Due to the limited bioavailability of ADCs—only approximately 2% reach the target tumor site following intravesical administration—the payload compounds must possess high toxicity, typically with IC50 values in the nM to pM range [18]. The efficacy of an ADC in delivering cytotoxic agents depends on three primary factors: the number of target cell surface antigens available for antibody binding, the internalization efficiency of the antigen-antibody complex, and the release of active cytotoxic agents from the intracellular antigen-antibody complex. Currently, ADC payloads exert their therapeutic effects primarily by disrupting microtubule polymerization and inducing deoxyribonucleic acid (DNA) damage [31]. This approach emphasizes the targeted and precise mechanism of ADCs in eliminating cancer cells.

#### Linkers

Cytotoxic payloads in ADCs are conjugated to the antibody via a linker, which must remain stable in vivo to ensure ADC integrity until it reaches the tumor cell. However, this linkage should be easily cleaved upon internalization. Maintaining ADC stability during circulation is essential to prevent premature payload release due to linker degradation, which could cause damage to healthy tissues. Simultaneously, ADCs must be capable of efficiently releasing their cytotoxic payloads once internalized by the target cells. Linkers are classified as cleavable or non-cleavable. Non-cleavable linkers include two main categories: thioether and maleimidocaproyl [32]. Cleavable linkers are further divided into three types: acid-cleavable, reducible disulfides, and those cleaved by exogenous stimuli [33]. Within the cleavable category, linkers can be potential of pH-sensitive, protease-sensitive, or glutathione-sensitive, based on their specific characteristics [34, 35]. Cleavable linkers are designed to break down in response to the distinct conditions of the tumor microenvironment, promoting the release of small molecule payloads. In contrast, non-cleavable linkers remain stable in both the bloodstream and tumor cells. When the ADC antibody is degraded in the lysosome, the small cytotoxic molecules, still attached to the linker, are released, exerting their anti-tumor effects [35].

### Development of ADCs

**Fig. 4:**
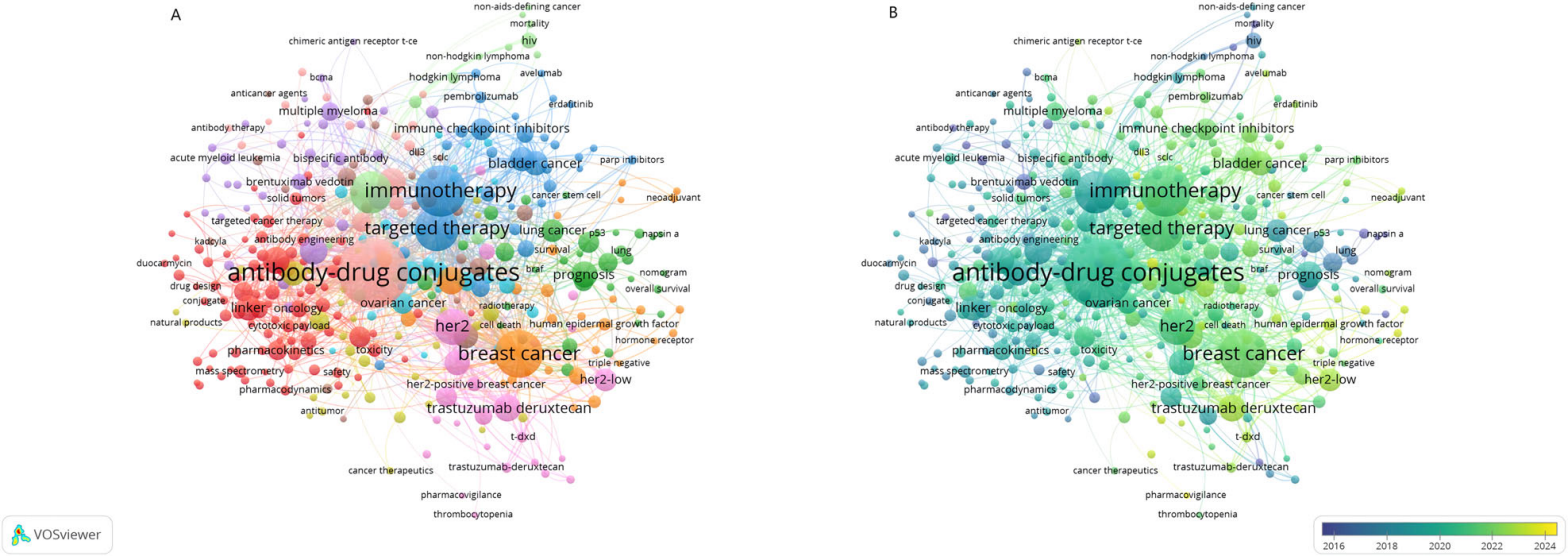
Term occurrence network visualization of ADCs. **A** The term clustering based on different field. **B** A chronological overview of terms based on 2016–2024. Each node in the map represents a term that occurred at least 5 times and the size of the node of a term is proportional to the number of occurrences of that term. The Core Collection of Web of Science (WOSCC) was used to retrieve and obtain relevant literatures on application of ADCs in tumor therapy since 2010–2024. All articles were retrieved on the same day to prevent partial results confusion due to rapid updates of subsequent publications.

ADCs are sophisticated constructs consisting of recombinant mAbs, cytotoxic agents, and linkers. Their development reflects significant advances in monoclonal antibody, payload, and linker technologies. The concept of ADCs was introduced by Paul Ehrlich in 1913 with his “magic bullet” theory. However, due to the technological limitations of that era, ADCs were first tested in animals in 1972, followed by human trials in 1983. A major breakthrough occurred in 1988 with the advent of humanized antibody technology, which mitigated the immunogenicity issues associated with murine antibodies, marking a pivotal moment in ADC development. Since then, the field has experienced rapid growth, and ADCs remain a dynamic focus of cancer therapeutic research [36]. Their application in oncology has garnered substantial interest and has led to widespread clinical utilization (Fig. 4).

Keywords are a fundamental component of academic articles, effectively capturing the central themes of a paper. In this study, all keywords from the retrieved publications were extracted and analyzed using VOSviewer software. A total of 369 keywords, each occurring at least five times, were included in the visualization. The size of the nodes reflects the frequency of the corresponding keywords, with larger nodes indicating higher frequency. The proximity between nodes represents the strength of the correlation between the terms, while nodes of the same color denote related topics (Fig. 4A). As expected, keywords associated with “antibody-drug conjugates” such as targeted therapy, immunotherapy, human epidermal growth factor receptor 2 (HER2), breast cancer (BC), and prognosis are prominently featured. Similar to the analysis of research themes, all keywords were categorized into 12 primary clusters.

Figure 4B illustrates the chronological evolution of keyword usage over time, derived from the analysis in Fig. 4A. Purple-marked terms indicate their predominant usage in earlier years, while green denotes those that have remained consistently relevant. For instance, terms like brentuximab vedotin and adenocarcinoma were frequently used prior to 2016, whereas keywords such as trastuzumab deruxtecan, disitamab vedotin, gold nanoparticles, and tumor microenvironment have gained prominence in recent years.

In 2000, the U.S. Food and Drug Administration (FDA) approved the first ADC, Mylotarg (gemtuzumab ozogamicin), for the treatment of adult acute myeloid leukemia, marking a significant milestone in the development of targeted ADC therapies for cancer [18]. However, post-approval studies revealed that Mylotarg was linked to fatal liver toxicity and failed to demonstrate a statistically significant improvement in overall survival (OS). As a result, the drug was withdrawn from the market in 2010 [37]. Despite this setback, Mylotarg remains a focus of ongoing scientific research. After further development, the FDA re-approved Mylotarg in 2017 [18, 38], reflecting the persistent efforts to refine and enhance ADC-based therapies for cancer treatment.

Advancements in ADC technology primarily aim to optimize antibodies, payloads, and conjugation methods to ensure targeted drug release at the tumor site, maximizing efficacy while minimizing off-target effects [39]. ADC efficacy is closely linked to the concentration of cytotoxic drugs within tumor cells [40]. The drug-antibody ratio (DAR), which represents the average number of cytotoxic molecules attached to each antibody, is a critical determinant of efficacy. Numerous studies have sought to enhance the DAR of ADCs [41]. However, while an increased DAR can improve efficacy, it may also exacerbate toxicity to normal tissues due to the polar nature of the cytotoxic agents [42, 43]. A DAR range of 3.5-4 is generally considered optimal, as values above 4 may result in suboptimal pharmacokinetic profiles and increased toxicity [44]. An exception to this is the development of an innovative cell-selective nanotoxin, based on anti-CD44 antibody-polymersome-DM1 conjugates (aCD44-AP-DM1), which achieves a DAR of up to 275 and promotes rapid, reductive release of DM1, offering potent treatment potential for solid tumors [45]. Consequently, precise determination and selection of the DAR are essential for optimizing ADC development, ensuring effective drug concentrations within tumor cells.

The technological development of ADCs has progressed through three distinct phases [18], reflecting their complex structure and function. The first generation primarily involved conventional chemotherapeutic agents linked to mouse-derived antibodies via non-cleavable linkers, unstable connectors, and random coupling methods. These early technologies resulted in ADCs with high immunogenicity, unstable DAR, increased risk of linker cleavage in the bloodstream, and premature release of cytotoxic drugs. Consequently, these ADCs exhibited high toxicity, severely limiting their therapeutic window. In the second generation, humanized antibodies were introduced, along with more stable linkers, although random coupling techniques were still employed. These improvements reduced the immunogenicity of the antibodies, lowering their clearance rate by the immune system and enhancing target binding. Furthermore, the stability of the linker in plasma was improved, leading to a notable increase in the therapeutic window. Despite these advances, issues such as off-target toxicity, a limited therapeutic window, and aggregation or rapid clearance of ADCs with high DAR persisted. The third generation introduced targeted coupling methods, resulting in greater molecular homogeneity and improved ADC stability. This technology also enhanced in vivo stability and adopted strategies focused on achieving high DAR with minimal toxicity. These innovations, characterized by reduced toxicity, enhanced anticancer activity, and improved stability, have significantly advanced the effectiveness of ADC-based therapies [46–48].

### Mechanism of action

**Fig. 5:**
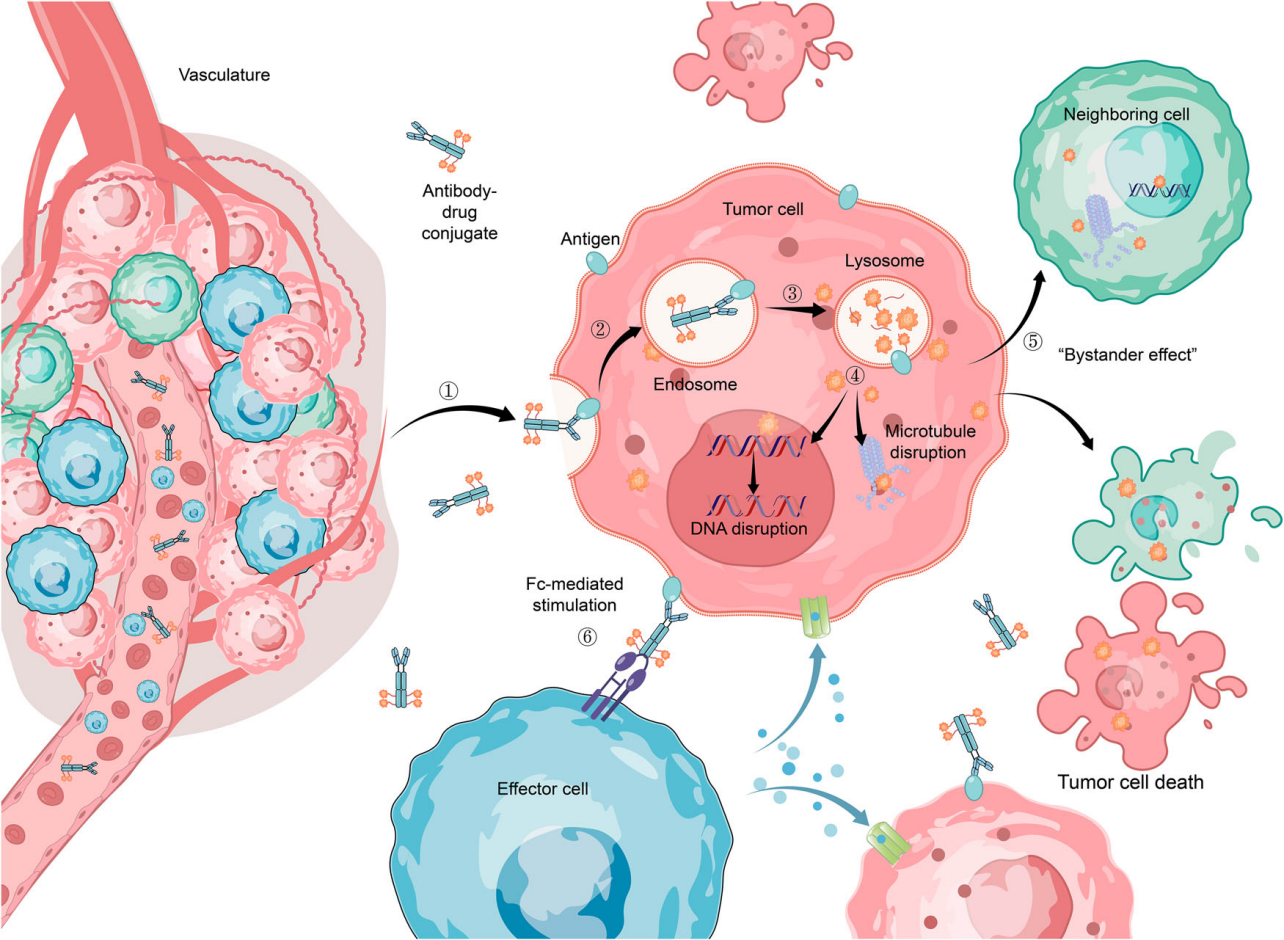
ADCs follow a defined mechanism of action: Initially, they circulate through the bloodstream to reach tumor tissues (①), where they bind to cell surface antigens. This interaction induces internalization of the ADC-antigen complex via antigen-mediated endocytosis (②), followed by processing within the endo-lysosomal pathway (③), which involves linker cleavage and potential antibody degradation. Once inside the cell, the drug payload is released. Depending on its type (④), such as microtubule-disrupting or DNA-targeting agents, it exerts its effects in distinct cellular compartments. Accumulation of the active drug ultimately triggers cancer cell death. Cytotoxic metabolites released during this process may diffuse to adjacent cells, contributing to bystander killing (⑤). Moreover, the Fc region of the antibody can enhance immune cell activity (⑥), promoting immune-mediated tumor cell destruction.

An ADC molecule enters tumor tissue via the vasculature, where it binds to a specific target on the tumor cell surface. This binding triggers internalization through the endosomal-lysosomal pathway. Within this pathway, the linker is cleaved, or the antibody is degraded, resulting in the release of the cytotoxic payload. The payload then diffuses into the cytoplasm and targets microtubule proteins, disrupting microtubule dynamics and inhibiting tumor growth. Certain payloads, such as calicheamicin, which target DNA, must further diffuse from the cytoplasm into the nucleus to exert their effects. If the cytotoxic metabolite is capable of passing through the cell membrane, it can diffuse into neighboring cells, inducing ‘bystander killing’. This process enhances the overall efficacy of the ADC by impacting not only the targeted cells but also surrounding tumor cells (Fig. 5).

### Limitations of ADCs

#### Pharmacokinetics

Pharmacokinetics examines the dynamic processes governing a drug’s absorption, distribution, metabolism (biochemical conversion), and excretion, with a particular focus on blood concentration over time [49]. Drug development must address both safety and efficacy considerations. ADCs, being complex pharmaceuticals composed of multiple components, pose significant challenges in research and development, particularly regarding their pharmacokinetic properties. Following administration, ADCs can exist in several forms within the body, including the intact ADC, naked antibodies resulting from linker degradation, and free payloads [50]. These forms continuously change as ADCs bind to their target antigens, undergo internalization, and subsequently dissociate. The concentration of intact ADCs and naked antibodies decreases after administration due to internalization and subsequent antibody clearance. This process is influenced by the mononuclear phagocyte system and Fc receptor (FcRn)-mediated recycling, where the FcRn binds ADCs in endocytic vesicles and transports them to extracellular spaces for reuse. As a result, antibodies, including both intact ADCs and naked antibodies, typically exhibit longer half-lives compared to conventional small-molecule drugs.

Free-carrier drugs are predominantly metabolized in the liver and eliminated through urine or feces. This metabolic process can be affected by drug-drug interactions, as well as conditions like hepatic or renal impairment [51, 52].

#### Drug resistance

Numerous studies [53–55] have highlighted the substantial benefits of ADCs in the treatment of various cancers. However, as research advances, drug resistance has become a significant obstacle that requires attention. Tumor cells can develop resistance mechanisms in response to any therapeutic intervention [56]. Resistance may manifest either after drug administration (secondary or acquired resistance) or at the onset of treatment (primary or denovo resistance).

##### Mechanisms underlying resistance to ADC therapies

Drug resistance during ADC therapy is influenced by multiple factors, such as downregulation of target antigen expression, modifications in ADC transport mechanisms, defects in internalization, overexpression of drug efflux transporters, and alterations in apoptotic signalling pathways [56].

One major factor is the downregulation or loss of target antigens, which can reduce ADC effectiveness. Mutations in the antigen that mAbs recognize, coupled with increased drug exposure, can significantly lower the expression levels of the target antigen. However, reduced drug exposure may enhance the efficacy of ADCs by preventing excessive antigen expression. Studies have shown that caveolin-1-mediated endocytosis of trastuzumab in HER2^+^ cell lines reduces drug sensitivity. This is because diminished antigen expression limits the efficient delivery of trastuzumab to the lysosome [57].

Autophagy also plays a critical role in modulating drug resistance through multiple mechanisms. ADC drugs release their cytotoxic payload via chemical or enzymatic cleavage within the lysosome, making the lysosomal environment a key player in ADC resistance [58]. The involvement of autophagy in cancer is complex. While autophagy functions as a tumour suppressor in early stages of cancer development, it also promotes tumour progression during carcinogenesis and in later stages of cancer [59–62]. Autophagy has been shown to increase cellular tolerance to drugs by lowering the levels of pro-apoptotic proteins, such as B-cell lymphoma-2 (BCL-2), while elevating anti-apoptotic proteins like BCL-2-associated X protein (BAX) [63]. Furthermore, prolonged exposure to trastuzumab leads to its accumulation in the lysosome, contributing to drug resistance. This resistance is associated with an elevated lysosomal pH, which inhibits the release of cytotoxins by lysosomal proteases [58].

Another factor is the overexpression of drug efflux transporters, which plays a significant role in ADC resistance. For instance, in HER2^+^ BC cells sensitive to trastuzumab, cyclin B levels, which are involved in the G2/M transition, are elevated compared to those in trastuzumab-resistant cells [64].

Finally, the upregulation of multidrug resistance genes can alter microtubule composition, resulting in reduced antigen expression, impaired antigen internalization by ADCs, and decreased intracellular trafficking or drug release [65].

##### Strategies to overcome ADC drug resistance

In response to the multifaceted causes of drug resistance outlined above, the scientific and medical communities must establish a collaborative intervention system encompassing drug development, clinical application, and public health management. To address these challenges, sciences propose the following multidimensional improvement strategies: optimizing antibodies targeting antigen mutations, optimizing linkers targeting lysosomal transport proteins, optimizing cytotoxins, optimizing multidrug resistance gene (MDR) pathways, and autophagy inhibitor co-administered with ADCs.

One potential solution to overcoming ADC resistance is optimizing the antibodies used, particularly through targeting antigen mutations. The use of bispecific and dual-epitope antibodies can improve the cellular uptake of ADCs. Bispecific ADCs bind two distinct, non-overlapping epitopes on the same target antigen, leading to enhanced receptor clustering and increased endocytosis, lysosomal transport, and degradation [66]. Compared to HER2-targeted ADCs, bispecific ADCs targeting both HER2 and prolactin receptors are more effective in killing cells co-expressing both receptors, suggesting that dual targeting promotes enhanced lysosomal endocytosis [67]. This approach addresses antigenic escape mechanisms while leveraging cooperative receptor dynamics.

Another key area of focus is improving the linkers that target lysosomal transport proteins. An increase in lysosomal pH causes trastuzumab to accumulate in the lysosome, disrupting its proteolytic activity. Many ADCs rely on the lysosomal internalization and degradation pathway to deliver their cytotoxic payloads. Thus, restoring lysosomal function could overcome ADC resistance and improve efficacy. Studies suggest that restoring the antitumour activity of trastuzumab and other ADCs can be achieved by modulating lysosomal pH, and some research proposes utilizing light-activated nanoparticles to manipulate lysosomal pH [58, 68].

Optimizing the cytotoxins is another approach. For example, novel payloads, such as pyrrolo[2,3-d] pyrimidine benzodiazepines [69], which bind to and cross-link specific DNA targets in cancer cells, can inhibit tumour cell proliferation without distorting the DNA helix, potentially reducing the risk of resistance development.

Addressing MDR gene pathways can also help overcome ADC resistance. Multidrug resistance genes exhibit a higher affinity for transporting hydrophobic compounds compared to hydrophilic ones. Developing hydrophilic or charged linkers may enhance the potency of ADCs against MDR^+^ cells with high polarity or charged metabolites. Singh et al. [70] designed ADCs using a novel trisaccharide peptide linker (CX), which cleaves a single peptide bond to release the cytotoxic payload within the lysosome. Compared to SMCC-linker ADCs, the CX-linker ADC demonstrated significantly higher cytotoxic activity, with an IC50 reduction of 5 to 100 times in multidrug-resistant cell lines. Tian et al. [71] developed a stable oxime bond linker between anti-5T4 and HER2 antibodies using MMAD and pacetylphenylalanine (pAcF), which improved both the stability and efficacy of the drug compared to conventional cysteine conjugates.

Autophagy inhibitors co-administered with ADC have also emerged as a promising strategy. For example, phosphoinositide 3-Kinase/Protein Kinase B (PI3K/AKT) inhibitors, such as Alpelisib, in combination with HER2-targeted therapies, can overcome drug resistance. Tian et al. [72] developed a stable oxime bond linker between anti-5T4 and HER2 antibodies using MMAD and pAcF, which improved both the stability and efficacy of the drug compared to conventional cysteine conjugates. A novel PI3K-p110α Proteolysis Targeting Chimera (PROTAC) has been developed, which effectively inhibits breast cancer cell proliferation by degrading PI3K-p110α and restores sensitivity to lapatinib. Furthermore, the clinical efficacy of the PI3K PROTAC exceeds that of Alpelisib, a selective PI3K-p110α kinase inhibitor [73]. The autophagy inhibitor LY294002 significantly enhanced the cytotoxicity of αCLDN18.2-MMAE and caspase-mediated apoptosis, highlighting the protective role of autophagy in CLDN18.2-targeted ADC-treated GC cells [62]. Co-administration with autophagy inhibitors markedly improved the in vivo antitumour efficacy of αCLDN18.2-MMAE. Additionally, combining ADCs with immune checkpoint inhibitors has yielded promising therapeutic outcomes in several clinical trials. One study proposed a novel treatment strategy by combining nectin-4 MMAE with autophagy inhibitors for bladder cancer therapy [74]. In contrast, autophagy inhibition by chloroquine suppressed Rituximab-MMAE-induced apoptosis, while autophagy activation by rapamycin significantly enhanced the therapeutic effect of Rituximab-MMAE in both in vitro and in vivo models [75].

These approaches may offer a pathway to more effective cancer therapies.

#### Toxicity

Several studies have identified key toxicities associated with ADCs in animal models, including hematopoietic stem cell depletion [76], hepatotoxicity [77], and reproductive toxicity [78]. Other reports highlight dermatotoxicity and nephrotoxicity [79]. Additionally, certain solid tumor cells may evade ADC targeting due to heterogeneous antigen expression, resulting in the “bystander effect” [31, 80, 81]. In some cases, antibody-induced toxicity is observed, as seen in the dermal toxicity linked to Pfizer’s trophoblast cell surface antigen 2 (Trop2) ADC, potentially caused by antigen expression in epithelial tissues [82]. However, in most instances, toxicities are primarily attributed to the cytotoxic payloads [83]. For example, microtubule inhibitors such as monomethyl auristatin E (MMAE) and monomethyl auristatin F (MMAF) can lead to neutropenia and thrombocytopenia, while kacinomycin is associated with liver injury. Due to their relatively large molecular size, ADCs tend to accumulate in tumor tissues [84], limiting their penetration into tumor cells and reducing therapeutic efficacy [85]. ADCs consist of an antibody conjugated to a payload via a linker [86], which ensures stability in the body and promotes controlled payload release within the lysosome of target tumor cells. Achieving this balance requires optimizing both the linker’s stability and the efficient cleavage of the payload from the ADC [87].

The combination of these factors, along with patient variability, complicates the development of a model to characterize the clinical features of ADCs, further increasing the challenges in designing novel ADCs. Additionally, molecular aggregation of ADCs, influenced by antibody structure, hydrophilic-hydrophobic interactions, and external environmental factors, has been shown to diminish the therapeutic efficacy of the drug [88]. Therefore, optimizing next-generation ADCs requires not only design improvements but also vigilant monitoring of adverse reactions during drug administration to mitigate potential risks.

## ADCs in GC treatment

### Target: HER2

HER2, also referred to as erythroblastic leukemia viral oncogene homolog 2 (ERBB2), is a distinct member of the ERBB family [89]. Unlike other ERBB receptors, HER2 lacks ligand-binding capacity and instead forms heterodimers with other ERBB family members. This interaction triggers the phosphorylation of intracellular tyrosine kinases, activating downstream signaling pathways [90].

HER2 plays a key role in regulating specific signaling pathways within the tumor microenvironment, which are essential for various cellular processes, including proliferation, apoptosis, migration, and angiogenesis [91, 92].

Through heterodimerization with HER3, HER2 activates the PI3K/AKT/mTOR pathway, promoting cell survival, metabolic reprogramming, and resistance to therapy [93, 94]. Additionally, HER2 activates RAS via the Grb2/SOS complex, which further stimulates the mitogen-activated protein kinase/extracellular rignal-regulated kinase pathway (MAPK/ERK) cascade, enhancing proliferation and epithelial-mesenchymal transition (EMT), thereby promoting metastasis [95, 96]. HER2 also activates the JAK/STAT pathway by inducing cytokines like interleukin-6 (IL-6), which in turn upregulates programmed death-ligand 1 (PD-L1), suppressing T-cell function and promoting cancer stem cell (CSC) self-renewal [97, 98]. Moreover, HER2 activates Src kinase, which drives cytoskeletal remodeling and extracellular matrix degradation, enabling cellular invasion [99, 100]. Activation of the nuclear factor kappa-B (NF-κB) pathway through PI3K/AKT or Src contributes to a pro-inflammatory microenvironment and the upregulation of anti-apoptotic proteins [101]. Additionally, HER2 stabilizes Wnt/β-catenin signaling by inhibiting GSK3β, reinforcing CSC maintenance and chemoresistance [102, 103]. Lastly, HER2 remodels the immune microenvironment by recruiting immunosuppressive cells, such as Tregs and M2 macrophages, and increasing PD-L1 expression, collectively promoting immune evasion [104, 105]. Therefore, targeting HER2 and its downstream signaling, in combination with immunomodulatory strategies, represents a promising therapeutic approach for HER2-positive GC.

Trastuzumab, in combination with chemotherapy and PD-1 checkpoint blockade, is emerging as the new first-line standard of care for HER2-positive advanced GC. Results from phase 3 clinical trials are eagerly awaited [106]. Recent years have seen substantial progress in the development of HER2-targeted therapies for GC.

#### RC48

RC48 is a novel third-generation anti-HER2 ADC composed of Hertuzumab, a high-affinity anti-HER2 monoclonal antibody with enhanced antibody-dependent cytotoxic activity compared to trastuzumab, conjugated to MMAE through a cleavable linker [107]. Upon internalization, RC48 selectively delivers MMAE to target cells [108], leading to antiproliferative effects by disrupting the HER2 signaling pathway and associated bypass pathways, including PI3K/AKT/mTOR and MAPK signaling [109]. This disruption results in G2/M phase cell cycle arrest via microtubule depolymerization and promotes apoptosis, thereby exerting antitumor effects [110]. RC48 has shown significant antiproliferative activity in HER2-positive cells. Given its robust clinical efficacy in HER2-positive GC, it was granted approval by the National Medical Products Administration (NMPA) in 2021 for the treatment of patients with locally advanced or metastatic gastric and gastroesophageal junction cancer (GC/GEJC) with HER2 overexpression, who have undergone at least two prior systemic chemotherapy regimens [111]. This approval was based on a single-arm, open-label, multicenter clinical trial enrolling 127 patients with HER2-overexpressing advanced GC and GEJC, who had previously received second-line or more chemotherapy regimens, such as vedolizumab. The trial reported an objective response rate (ORR) of 23.6%, a median progression-free survival (mPFS) of 4.1 months, and a median OS of 7.6 months (NCT02881190) [112].

#### T-DM1

Trastuzumab emtansine (T-DM1) is an ADC composed of trastuzumab, the anti-mitotic microtubule inhibitor DM1, and a thioether-based linker, maleimide methyl cyclohexanecarboxylate [113]. It is FDA-approved for the treatment of HER2-positive advanced BC [114]. This approval opens the possibility for further exploration of T-DM1 as a targeted delivery system in GC. T-DM1 specifically delivers the apoptosis-inducing agent DM1 to HER2-positive tumor cells, functioning as a “precision missile.” Studies [115] have demonstrated that T-DM1 exhibits robust antitumor activity against HER2-positive GC cells both in vitro and in vivo, including efficacy against trastuzumab-resistant tumors. However, a randomized, open-label, multicenter phase II/III trial [116] found that T-DM1 did not show superiority over paclitaxel in previously treated HER2-positive advanced GC patients. In response, Seo et al. [117] experimentally revealed that a substantial proportion of HER2-positive patients underwent HER2-negative transformation and increased genetic heterogeneity following chemotherapy with trastuzumab-based regimens. This downregulation of HER2 expression highlights the importance of reassessing HER2 status prior to initiating second-line anti-HER2 therapy.

#### DS-8201a

Trastuzumab Deruxtecan (DS-8201a) [118], also known as T-DXd, is a second-generation ADC consisting of trastuzumab, a cleavable tetrapeptide linker, and the topoisomerase I inhibitor, DXd. Unlike T-DM1, which has a DAR of 3.5, T-DXd features a distinct DAR of 8, owing to its unique tetrapeptide linker (GGFG) [119]. This linker is selectively cleaved by lysosomal enzymes within tumor cells, ensuring the targeted release of the drug while minimizing impact on the surrounding circulation [120]. Upon binding to the HER2 receptor, T-DXd disrupts HER2 signaling and induces an antibody-dependent cellular cytotoxicity (ADCC) response [121]. Additionally, T-DXd is internalized via endocytosis, undergoes cleavage within the lysosome, and releases the free DXd payload, resulting in DNA damage and subsequent apoptosis [122]. In experiments using the HER2-positive GC cell line (NCI-N87) and a T-DM1-resistant variant (N87-TDMR) developed through continuous exposure to T-DM1, the efficacy of DS-8201a was assessed, suggesting its potential for treating T-DM1-resistant patients with BC or GC. Phase I clinical trials evaluating DS-8201a’s therapeutic efficacy and safety have demonstrated its promising anti-tumor activity and manageable safety profile. As a result, the FDA approved DS-8201a in 2021 for use in patients with locally advanced or metastatic HER2-positive GC/GEJC who have previously undergone trastuzumab-based treatments.

The ongoing DESTINY-Gastric 06 trial seeks to validate the survival benefits of DS-8201a in Chinese patients [123]. Currently, the standard second-line treatment for advanced GC consists of a combination of ramucirumab and paclitaxel. To explore more effective alternatives, the DESTINY-Gastric 04 trial is also underway. Ogitani et al. [124] reported anti-tumor activity in a patient-derived xenograft (PDX) model of BC with low HER2 expression. Additionally, a phase I pilot study assessing dosage toxicity in GC patients with low HER2 expression demonstrated significant antitumor effects. These results highlight the potential of this therapy for GC patients with low HER2 expression [125]. T-DXd showed remarkable efficacy and an acceptable safety profile, owing to its unique DXd payload and stable linker.

#### ARX788

ARX788 [126] is a novel ADC comprising an anti-HER2 mAb conjugated to the microtubule inhibitor AS269. This ADC is site-specifically coupled to the antibody using unnatural amino acids, resulting in high drug homogeneity and a relatively low DAR of approximately 2 [127]. Once internalized by tumor cells, ARX788 releases amberstatin, which induces cell cycle arrest and apoptosis, thereby exerting anticancer effects. The non-cleavable linker, coupled with pAcF, allows ARX788 to mitigate the non-targeted toxicity observed in previous generations of ADCs. ARX788 has demonstrated efficacy against trastuzumab-resistant GC cells [128]. In PDX models with high and low HER2 expression, treatment with ARX788 and T-DM1 showed that ARX788 exhibited stronger anticancer activity compared to T-DM1 [129]. Furthermore, ARX788 displayed high serum stability and a relatively long half-life (~12.5 days) in mice. Skidmore et al. [127] demonstrated in vitro that ARX788 was more effective in cell lines with low HER2 expression than T-DM1, while showing no activity in normal cardiomyocytes. Additionally, ARX788 significantly inhibited tumor growth, outperforming T-DM1 in BC with high HER1 and low HER2 expression, as well as in GC-derived xenografts.

A recent phase I randomized, open-label, international multicenter trial [128] assessed the efficacy of ARX788 in 30 patients with advanced GC. Compared to trastuzumab monotherapy, the combination of ARX788 and trastuzumab significantly improved OS, with an ORR of 37.9% [95% CI (20.7%, 57.7%)] and a disease control rate (DCR) of 55.2% [95% CI (35.7%, 73.6%)]. At a median follow-up of 10 months, the mPFS and OS were 4.1 months [95% CI (1.4, 6.4)] and 10.7 months [95% CI (4.8, not reached)], respectively, while the median duration of remission was 8.4 months [95% CI (2.1, 18.9)]. These results suggest that ARX788 demonstrates promising antitumor activity in patients with HER2-positive advanced gastric adenocarcinoma. Currently, a phase II/III international, multicenter, randomized controlled study is underway to further evaluate the efficacy and safety of ARX788 in second-line treatment for HER2-positive advanced GC/GEJC adenocarcinoma. This study provides valuable data and a theoretical foundation for subsequent clinical trials of this novel HER2-targeted ADC.

#### MRG002

MRG002 is an ADC consisting of sugar-modified trastuzumab conjugated to MMAE via a valine-citrulline linker, with a DAR of 3.8 [130]. It exhibits a similar affinity for HER2 as trastuzumab but has reduced ADCC activity and minimal effects on immune cells due to the high fucosylation of its mAb component. In animal studies, MRG002 has been shown to induce tumor regression in a CDX model with low HER2 expression [130, 131]. Preclinical data further demonstrate solid anti-tumor activity in mammary and gastric xenograft models with varying HER2 expression levels. MRG002 also outperformed both trastuzumab and T-DM1 monotherapy in mouse xenograft models. Additionally, MRG002 significantly enhanced the anti-tumor effects of anti-PD-1 mAb, suggesting a potential combination therapy for GC patients [131]. A phase I trial of MRG002 as a monotherapy (NCT04941339) is currently underway in patients with HER2-positive relapsed or refractory GC. Meanwhile, several phase II trials are evaluating MRG002’s efficacy in treating various malignancies with HER2-positive or low-HER2 expression, including an ongoing study in locally advanced or metastatic GC/GEJC with low HER2 expression [132].

#### Others

Legochem Biosciences’ ADC (LCB-ADC) represents a novel class of HER2-targeted ADCs, composed of trastuzumab conjugated to MMAF via a sophisticated cleavable linker [133]. n contrast to T-DM1, all LCB-ADCs incorporate cleavable linkers engineered to enhance anticancer activity through multiple mechanisms. Additionally, LCB-ADC demonstrates a more potent ADCC effect and a higher G2/M phase arrest rate compared to T-DM1 [133]. It also exhibits significant anti-tumor efficacy. PF-06804103, another HER2-targeted ADC, offers a broader therapeutic window and extended indications relative to T-DM1. Moreover, it presents favorable pharmacokinetic properties, enhanced stability, and reduced off-target toxicity [134]. A comprehensive clinical evaluation of LCB-ADCs in patients with GC is warranted.

Alongside these agents, several other novel HER2-targeted ADCs, including XMT-1522 [55], SYD985 [135], BAT8001 [136], ZW49 [137], are undergoing clinical trials. The efficacy and safety of these therapies for patients with low HER2-expressing GC have attracted considerable interest (Table 2).

**Table 2:** Key clinical trials of HER2-targeted ADCs in gastric cancer.

Trial number	ADC	Stage	Patients	mOS	ORR	mPFS	Adverse	Refs
NCT03556345	RC48	II	127	7.9 months	24.80%	5.5 months	Decreased WBC count: 53.6%; asthenia: 53.6%; hair loss: 53.6%; decreased neutrophil count: 52.0%; anemia: 49.6%; increased ASP SAEs: 36.0%; aminotransferase level: 43.2%.	[110]
NCT02881190	RC48	I	57	7.6 months	23.60%	4.1 months	Neutropenia: 19.3%; leukopenia: 17.5%; hypoesthesia: 14.0%; conjugated blood bilirubin increased: 8.8%.	[112]
NCT01641939	T-DM1	II/III	204	7.9 months	20.60%	2.66 months	Alopecia: 51.35%; peripheral neuropathy: 19.82%; thrombocytopenia: 14.41%.	/
NCT02318901	T-DM1	I/II	16	/	/	/	Thrombocytopenia: 7.25%; fatigue: 19.64%; peripheral neuropathy: 9.38%.	/
NCT03329690	DS-8201a	II	233	8.4 months	/	3.5 months	Decreased neutrophil count:51% vs. 24%; anemia: 38% vs. 23%; decreased white cell count: 21% vs. 11% (DS-8201a vs. chemotherapy)	[123]
NCT03329690	DS-8201a	II	233	7.8 months	26.30%	7.8 months	Anemia: 30.0% and 29.2%; decreased neutrophil count:25.0% and 29.2%; decreased appetite: 20.0% and 20.8% in cohort 1 and 2.	[174]
NCT04014075	DS-8201a	II	79	/	42%	5.5 months	SAEs: 41.77%.	[175]
NCT03255070	ARX788	I	106	10.7 months	37.90%	4.1 months	AEs: 93.3%.	[128]
NCT02277717	SYD985	I	185	/	/	/	/	[135]

*ADCs* antibody-drug conjugates, *mOS* median overall survival, *mPFS* median progression-free survival, *ORR* objective response rate, *Refs* references, *WBC* white blood cell, *SAEs* serious adverse events, *AEs* adverse events, *I/II/III* cancer stage I/II/III.

### Target: GCC

Guanylate cyclase C (GCC) is a receptor for heat-resistant enterotoxins that induce diarrhea, as well as for the endogenous ligands guanosine and uroguanosine [138]. It is primarily expressed in the intestinal epithelium [139]. GCC plays a key role in regulating intestinal fluid and ion secretion, as well as inhibiting cell proliferation. Research has shown that, compared to normal tissues, GCC is overexpressed on the surface of malignant tumors, including those in peripheral blood and the intestine. Consequently, GCC is gaining attention as a potential target for ADCs [140].

#### TAK-164

TAK-164 is an ADC targeting glycoprotein-coupled receptors (GCC receptors) in colorectal cancer (CRC) and GC [141, 142]. The construct consists of a 5F9 antibody backbone conjugated to a DNA-alkylating DGN-549 payload. Bruna examined the impact of coadministration of 5F9 on the distribution and efficacy of TAK-164 in a human tumor xenograft mouse model. Both experimental and computational data indicate that the observed effects were not attributable to tumor saturation, increased binding to perivascular cells, or compensatory bystander effects. Instead, the cellular potency of DGN-549 was aligned with the single-cell uptake of TAK-164, resulting in an IC50 close to its equilibrium binding affinity [143]. In a separate clinical trial [144], 31 patients with GCC-positive, advanced gastrointestinal cancers received intravenous TAK-164 on day 1 of 21-day cycles. No dose-limiting toxicities (DLTs) were observed during the evaluation period for cycle 1. However, following the second cycle, three patients experienced dose-limiting treatment-emergent adverse events, including grade 3 pyrexia and grade 5 hepatic failure (0.19 mg/kg), grade 4 hepatic failure with a reduction in platelet count (0.25 mg/kg), and grade 3 nausea along with grade 4 reductions in platelet and neutrophil counts (0.25 mg/kg).

TAK-164 demonstrated a manageable safety profile at 0.064 mg/kg, although hepatic toxicity was identified as a potential risk. The recommended phase 2 dose (RP2D) of 0.064 mg/kg was deemed insufficient to achieve clinical benefit, and no further clinical development was planned (NCT03449030).

#### TAK-264

TAK-264 is a novel ADC targeting GCC, comprising an IgG1 monoclonal antibody, the cytotoxic payload MMAE, and a protease-cleavable linker (peptide maleimide-hexanoyl-valine-citrulline). Preliminary animal studies have shown promising results in a high-expression GCC model, where TAK-264, via its protease-cleavable linker, delivers the potent anti-microtubule agent MMAE (linker and toxin licensed from Seattle Genetics) [145]. Clinical trial data [146] revealed that the most common side effects of TAK-264 were malignancy (41% of cases), loss of appetite (41%), fatigue (32%), and diarrhea (27%). The incidence of secondary adverse effects, including neutropenia and hypokalemia, was 22% and 7%, respectively, suggesting that TAK-264 is generally well tolerated. Among the 41 patients enrolled in the trial, one achieved partial remission, three had stable disease, and the mPFS was 44 days. These findings indicate that TAK-264 has limited efficacy in advanced GC patients but demonstrates a manageable safety profile. Preliminary evidence also suggests potential antitumor activity in specific gastrointestinal malignancies. Given the high expression of GCC in GC, this target remains a promising approach for the treatment of advanced GC. A further study [147] supports these results, indicating a similar outcome.

### Target: HER3

The HER3 protein, a member of the HER receptor tyrosine kinase (RPTK) family, is expressed in various solid tumors. Due to its role in promoting cell proliferation, targeting HER3 could have clinical significance. It is now recognized that the activation of HER3’s downstream signaling pathway plays a key role in modulating the therapeutic sensitivity of different tumor types. Overexpression of HER3 in GC patients is associated with poor prognosis.

#### EV20/NMS-P945

EV20/NMS-P945 is an ADC composed of EV20, the thienylindole derivative NMS-P528, and a cleavable linker, with an average DAR of 3.6. This ADC demonstrates favorable stability and a prolonged terminal half-life [148]. EV20/NMS-P945 exhibits target-dependent cytotoxicity in vitro against a range of cancers, including pancreatic, prostate, gastric, ovarian, and melanoma cell lines. Additionally, it shows promising anticancer activity in a mouse xenograft tumor model [148]. The observed effectiveness may be attributed to the thienoindole derivative NMS-P528, which is highly active in heterogeneous tumors. This activity is enhanced by the bystander effect of the toxin in cancer cells with low cell doubling times, making it a promising therapeutic option for solid tumors. These results suggest that EV20/NMS-P945 may serve as an effective therapeutic strategy for targeting HER3.

#### U3-1402

U3-1402 consists of a humanized anti-HER3 IgG1 mAb conjugated to a large topoisomerase I inhibitor payload, an Ezetimibe derivative of DXd, through a cleavable tetrapeptide linker [149]. This ADC offers several advantages, including high specificity, low toxicity, extended duration of action, and potent activity. It binds to HER3, which is overexpressed on the surface of tumor cells, forming the HER3-U3-1402 complex. This complex is then internalized into the endosome and subsequently processed by lysosomes. Inside the lysosome, a histone protease cleaves the linker, releasing the topoisomerase I inhibitor DXd, which induces DNA damage and impairs repair mechanisms [150].

In preclinical studies, U3-1402 demonstrated superior activity compared to the pattumab monoclonal antibody, showing therapeutic efficacy in cell lines resistant to first-generation tyrosine kinase inhibitors and in mouse models. Treatment was initiated when tumor volumes ranged from 80 to 250 mm³, and as expected, U3-1402 significantly inhibited tumor growth compared to vehicle treatment [151]. Data from a phase I clinical trial (NCT03260491) in patients with advanced non-small cell lung cancer reported a 100% disease control rate [152]. Two ongoing clinical studies investigating HER3-positive BC have shown promising survival benefits, with ORR of 33% and 42.9%, respectively [153, 154]. .

In addition to U3-1402, several other HER3-targeted ADCs, including MCLA-128 [155], MM-121 [156], and CDX/KTN3379 [157], are currently under investigation in clinical trials. The results of these studies may offer hope for GC patients in the near future.

### Target: CLDN18.2

Claudin 18.2 (CLDN18.2) is a key structural protein involved in tight junctions between cells [158]. While it is minimally expressed in normal tissues, it is highly upregulated in malignant tumors such as GC, pancreatic cancer, and esophageal cancer, positioning it as a promising target for tumor therapy. Recent advancements in CLDN18.2-targeted therapies have shown significant progress [159, 160]. Furthermore, emerging research suggests that CLDN18.2-positive GC may exhibit a distinct immune microenvironment [161]. Combining CLDN18.2 targeting with immune activation may offer a synergistic therapeutic effect, potentially enhancing treatment outcomes in patients with advanced GC.

#### CMG901

CMG901 is an ADC consisting of a humanized monoclonal antibody, CM311, targeting CLDN18.2, and the microtubule polymerization inhibitor MMAE, which is linked via a cleavable linker arm. The monoclonal antibody component of CMG901 selectively binds to CLDN18.2-positive cells, and upon binding, the ADC is internalized by tumor cells through endocytosis. Once inside the cell, the cleavable linker releases the cytotoxic MMAE payload within lysosomes, resulting in tumor cell apoptosis [162]. CMG901 (AZD0901) has been approved by the FDA for a phase I clinical trial (NCT04805307) targeting advanced solid tumors, including gastric and pancreatic cancers, where it demonstrated an ORR of 33% and a DCR of 70% in 89 patients [163].

#### Others

EO-3021 (SYSA1801) demonstrated efficacy in 17 GC patients (NCT05009966), with an ORR of 47.1% and a DCR of 64.7%. Preliminary evidence has emerged from the ongoing clinical trial of SOT102 (NCT05525286) that suggests the drugs may be effective in treating patients with GC/GEJC. However, it should be noted that the associated trial have been paused for the time being.

In summary, CLDN18.2 is characterized by high expression specificity in tumor tissues, making it a promising target for drug development and potentially transforming the treatment landscape for GC [161]. The molecular profiling of GC patients using multi-omics data can further explore the therapeutic potential of targeting CLDN18.2 in this context. Additionally, further clinical studies are required to determine the optimal dosing and effective combination strategies for targeted CLDN18.2 therapy.

### Target: TROP2

TROP2, a cell surface glycoprotein encoded by the TACSTD2 gene, is integral to regulating tumor growth by modulating various signaling pathways involved in tumor invasion, proliferation, and progression [164]. As a result, TROP2 has gained significant attention as a therapeutic target, with ADCs targeting TROP2 currently under investigation. However, no TROP2-targeted ADCs have been approved for clinical use to date.

#### SKB264

SKB264 is a novel ADC composed of a humanized monoclonal anti-TROP2 antibody linked to a topoisomerase I inhibitor via a cleavable linker [165]. This design provides several advantages, including the specificity of the monoclonal antibody for tumor cell antigens and the high efficacy of the cytotoxic agent. Preliminary results from the international multicenter Phase I-II first-in-human trial of SKB264 (NCT04152499) indicated that 17 patients were assessed for efficacy, with an overall ORR of 35.3%, a DCR of 70.6%, and a partial response observed in one patient with GC. These initial findings suggest that SKB264 is effective in treating solid tumors, and the ongoing Phase II expansion study aims to explore additional indications. The potential benefits of this treatment include providing relief for patients with locally advanced, unresectable, or metastatic solid tumors resistant to standard therapies.

#### IMMU-132

IMMU-132 is a novel ADC developed by coupling a humanized anti-TROP2 monoclonal antibody (hRS7) with the active irinotecan metabolite SN-38 [164]. A Phase I/II clinical trial (NCT01631552) demonstrated its antitumor efficacy in patients with TROP2-overexpressing gastric and pancreatic cancers. Additionally, IMMU-132 was shown to be more selective and safer than irinotecan, with a reduced incidence of diarrhea [166]. However, further investigation is required to fully assess the efficacy of IMMU-132 in TROP2-positive advanced solid tumors.

### Target: Nectin-4

Nectin-4 (gene name PVRL4) is a member of the Nectin family within the immunoglobulin superfamily of proteins, with high expression in breast, ovarian, and gastric cancers. It is closely associated with tumorigenesis and metastasis [167]. Nectin-4 promotes cancer cell proliferation and metastasis by activating the PI3K/AKT pathway and EMT-related signaling [168, 169]. Studies have shown that messenger ribonucleic acid (mRNA) and protein expression of Nectin-4 is elevated in GC tumor tissues compared to adjacent normal tissues, and its positive expression correlates with the OS of GC patients [170]. These results suggest that Nectin-4 plays a significant role in GC development and may serve as a potential clinicopathological biomarker and therapeutic target in GC [171, 172].

#### Nectin-4 NDC

Trivalent Nanobody (Nb), with a molecular weight of 42 kDa, consists of two homologous Nectin-4 Nbs and a high-affinity Nb (15 kDa) targeting human serum albumin [173]. This Nb is conjugated to a microtubule inhibitor, MMAE, via maleimide linkers containing valine-citrulline cleavage sites, resulting in a DAR of 1. Trivalent Nb overcomes the limitations of traditional IgG-based ADCs by leveraging the unique advantages of nanoantibodies, including enhanced tumor tissue penetration and more uniform targeted binding. Significant tumor suppression was demonstrated in an in vivo model. Cytotoxicity assays using the cell counting kit-8 method showed that Nectin-4 nanobody-drug conjugates (NDC) inhibited growth by approximately 60% in the NCI-N87 cell line, and by 70% at nanomolar concentrations in the 293T-human-Nectin-4 cell line. Histological and immunohistochemical analyses revealed that tumor tissues treated with Nectin-4 NDC exhibited pronounced morphological alterations, including vacuolization, nucleolysis, reduced cell density, and disrupted cellular structure, suggesting that Nectin-4 NDC effectively induces apoptosis in gastric adenocarcinoma cells [173].

In summary, the advancement of ADC therapeutics heralds a transformative era in GC management. ADCs exhibit a fundamental distinction from conventional chemotherapy, undergoing continuous evolution and refinement. The paradigm of targeted delivery, potent therapeutic efficacy, and favorable safety profiles inherent to ADC therapeutics will persist and be further optimized (Table 3). With ongoing technological innovations, ADC platforms are anticipated to deliver substantial clinical benefits to an expanding patient population.

**Table 3:** Composition of ADCs targeting gastric cancer.

NCI thesaurus code	ADC	Therapeutic target	Antibody	Payload	Linker	DAR
C169918	RC48	HER2	Hertuzumab	MMAE	VC	4
C82429	T-DM1	HER2	Trastuzumab	DM1	SMCC	3.5
C128799	DS-8201a	HER2	Trastuzumab	DXd	Tetrapeptide	8
C123917	ARX788	HER2	Anvatabart	Amberstatin269	pAcF	1.9
C174205	MRG002	HER2	Trastuzumab	MMAE	VC	3.8
C172821	LCB-ADC	HER2	Trastuzumab	MMAF	Undisclosed	2
C132112	XMT-1522	HER2	HT-19	AF-HPA	Fleximer polymer	12
C118674	SYD985	HER2	Trastuzumab	Duocarmycin	VC	2.8
C173966	BAT8001	HER2	Trastuzumab	Maytansine	6-Maleimidocaproic acid	/
C162115	ZW49	HER2	ZW25	N-acyl	Protease	2
C101524	TAK-264	GCC	Indusatumab	MMAE	VC	7.4
C156707	TAK-164	GCC	5F9	DGN549	Peptidic	/
[150]	EV20/NMS-P945	HER3	EV20	NMS-P945	Peptidic	3.6
C136987	U3-1402	HER3	Patritumab	DXd	Tetrapeptide	8
C180413	CMG901	CLDN18.2	mAb	MMAE	Undisclosed	8
C190788	SOT102	CLDN18.2	IgG1 mAb	PNU-159682	Amide/peptide	2
C102783	IMMU-132	TROP2	hRS7	SN-38	CL2A	7.6
C166409	SKB264	TROP2	Sacituzumab	T030	CL2A	7.4
[173]	Nectin-4 NDC	Nectin-4	Nb	MMAE	maleimide	1

*HER* human epidermal growth factor receptor, *TROP2* trophoblast cell surface antigen 2, *GCC* Guanylate cyclase C, *MMAE* monomethyl auristatin E, *MMAF* monomethyl auristatin F, *AF-HPA* auristatin f-hydroxypropylamide, *VC* valine-citrulline, *DM1* mertansine, *DXd* deruxtecan, *SMCC* succinimidyl-4-(Nmaleimidomethyl) cyclohexane-1-carboxylate, *pAcF* para-acetyl-phenylalanine, *IgG* immunoglobulin G, *NDC* nanobody-drug conjugate, *Nb* nanobodies.

## Conclusions and perspectives

ADC therapy has emerged as a promising treatment option for cancer, with several foundational and early-phase clinical trials for digestive system tumors currently underway. These trials have highlighted the unique potential of ADCs in the treatment of GC. Nevertheless, the development of ADCs faces several challenges, including the complexity of the drug structure, targeted delivery, and associated toxic side effects.

Key issues requiring urgent focus include overcoming drug resistance and understanding the mechanisms underlying antigenic expression heterogeneity. The continued research and development of ADCs must be underpinned by comprehensive clinical data and accurate disease classification to ensure their effective integration into clinical practice. Despite ongoing efforts, challenges persist, leading to suboptimal results in clinical trials, suggesting that significant progress is still needed to meet the original therapeutic objectives.

Looking forward, the development of next-generation ADCs, particularly those utilizing recombinant drug strategies, is essential to enhance cancer cell targeting and lysosomal delivery. For example, antibodies conjugated with cell-penetrating peptides may improve ADC targeting and facilitate lysosomal internalization. Future ADC advancements will require the creation of more precise humanized mAbs, more potent cytotoxic payloads, stable, enzymatically cleavable linkers, and innovative coupling technologies. These innovations are expected to optimize therapeutic efficacy, minimize off-target toxicity, and broaden the clinical applications of ADCs. Additionally, emerging technologies, including alternatives to mAbs such as nanobodies and protein scaffolds, hold promise for advancing ADC development.

Clinical and translational strategies are essential for expanding the therapeutic window of ADCs. Moreover, combination therapies have the potential to enhance ADC efficacy and mitigate resistance. With ongoing optimization and insights gained from two decades of clinical experience, the development of more efficient and safer ADCs for GC treatment is expected, ultimately leading to improved patient survival.
